# Supplementary material for: The Dual Prey-Inactivation Strategy of Spiders—In-Depth Venomic Analysis of Cupiennius salei
Source: Toxins (Basel). 2019 Mar 19;11(3):167. doi: 10.3390/toxins11030167 (PMC6468893; doi:10.3390/toxins11030167)
Supplement: Supplementary file 1 [file toxins-11-00167-s001.zip › Supplementary Fig S1.1-S1.19/Supplementary Fig S1.8.pdf]

Supplementary Fig S1.8

CLUSTAL O(1.2.4) multiple sequence alignment: CRISP1s

```
CRISP1_a1_CUPSA         MSAPARIRNILLVL----SSVLCTLTVIQSAYCPDIYKRYSTQHSYCLGQCCNCTYYKRG
CRISP1_b1_CUPSA         MSAPARIRNILLVL----SSVLCTLTVIQSAYCPDIYKRYSTQHSYCLGQCCNCTFYKRG
sp_A9QQ26_LYCSI         MSAPVGIPSLLLAL----CALLCVLNAVRSAYCPDPYKRYSDQHSYCVGVCCSCAKYRRG
CRISP1_VIRFA            ------IRSITLSFIVLLSAAVTIYAQELWGPCPDKYKRYNDMHSYCLRPCCSCAPYKRD
tr_A0A087T5V6_STEMI     MV---------NHVIRIGLFFVLLLGLSECAKC--PYAKFSPDHSFCKDPNPKCTILERG
tr_A0A087T5V4_STEMI     MA---------AGVITIISAIALAFGFIECGRC--PYDKFTPNHSFCKPPNPSCNILERG
                                     .                .  *   * ::.  **:*     .*   .*.

CRISP1_a1_CUPSA         VDPGLKGQILSLHNDLRNKVAAGHSYGVDHLPRASNMLEMVWDDELATIAQTWLDGCR-K
CRISP1_b1_CUPSA         VDPGLKGQILSLHNDLRNKVAAGHSYGVDHLPRASNMLEMVWDDELATIAQTWLDGCR-K
sp_A9QQ26_LYCSI         VSPWLKKELVRLHNNLRSKVAGGKSHSVDHLPRATNMLEMVWDDELAEVAQRWSDKCE-R
CRISP1_VIRFA            VSEGLKNYILFLHNSYRSKVAGGHSFGVNHLSRAANMLEMVWDDELAQIAQKWAYSCPSS
tr_A0A087T5V6_STEMI     LKSDDKQRLLDLHNMYREKVASGKETQAGKLPTATNMFEMAWDDELASIAQKWAEQCI-Y
tr_A0A087T5V4_STEMI     VGAGDRMKILKLHNDYRVRVAAGQETEAGGLPPAANMLEMVWDDELAAVAQKHAEQCN-F
                        :    :  :: ***  * :**.*:.  .. *  *:**:**.****** :**     *

CRISP1_a1_CUPSA         NGDCEECRMVSRFAVGQEIIGDFIGSEMDSQDLDNEFFSRFNALSVFTDAHVREYSDSNG
CRISP1_b1_CUPSA         NGDCEECRMVSRFAVGQEIIGDFIGSEMDSQDLDNEFFSRFNALSVFTDAHVREYSDSNG
sp_A9QQ26_LYCSI         KSDCEDCRRVGRFGVGQIIFE--FDGKMDGKDMENEFFSRFMALQSFRKEQVARFTA-GT
CRISP1_VIRFA            KSDCDLCRMVDRFPVGQLVIE--YPGKMDEKDLEMEFENFFNGLGSVKKEHLSSFNAADG
tr_A0A087T5V6_STEMI     DHDCDECRQVDRFDVGQNLYRAGSSDKPTKHDWDRMVKKWYDEVSMFDKSYIKPFFS-GV
tr_A0A087T5V4_STEMI     QHDCSDCRRVERFRVGQNIYIAMNSEPPKKQDWDRVIKDFYDEVSTFNRNYIKPFVF-GS
                        . **. ** * ** *** :           :* :  . . :  :  .    :  :   .

CRISP1_a1_CUPSA         NHKTSQLLWAKTWRIGCGFLRFLDPNNGRTVVNILLCNYGPGGCVEGEEVYKAGTVCSAC
CRISP1_b1_CUPSA         NHKTSQLLWAKTWRIGCGFLRFLDPNNGRAVVNILLCNYGPGGCVEGEEVYKAGTVCSAC
sp_A9QQ26_LYCSI         NPKTAQILWAKTWRIGCGFLDFNYPGNGRRYT-QIVCNYGPKGNEEGEEVYKAGSVCSSC
CRISP1_VIRFA            KGGYARLLWAKTWRIGCGFLYYNSNQ---KKKSSLVCNYGPAGNIEGEEVYKAGDACSAC
tr_A0A087T5V6_STEMI     YNHFSQIVWATTWKLGCGFVVYKEPE---WYRSYLVCNYGPTGNWDEEEMYNVGRTCSYC
tr_A0A087T5V4_STEMI     YGHFTQVVWAETWKVGCGYVLYKDPQ---WYNSFFVCNYGPAGNWMDGEMYKAGKTCSAC
                            ::::** **::***:: :            ::***** *     *:*:.* .** *

CRISP1_a1_CUPSA         PANTCCGDGCKK-HKITNSFKGLCKVIDENLPPQGNVPHVKSGKELFYCGFNGESDCSRA
CRISP1_b1_CUPSA         PANTCCGDGCKK-HKITNSFKGLCKVIDENLPPQGNVPHVKSGKELFYCGFNGESDCSRA
sp_A9QQ26_LYCSI         PANTCCGDACKK-HNLKANYHGLCKVIDENLPPEGNVPHKKTGNEVFYCGFNDESDCRHT
CRISP1_VIRFA            PGNTCCGKDCKN-HGTEAKYKGLCKVIDVNKPPEGDVPHQKTGKEIFYCGFNGESDCSYT
tr_A0A087T5V6_STEMI     PTGSCCGADCTKKFKKTAKYPSLC------------------------------------
tr_A0A087T5V4_STEMI     PEGTCCGADCSRKYNVIPKYSKLCMVVKGN------------------------------
                        * .:***  *.. .    .:  **

CRISP1_a1_CUPSA         VEGINRWIGKISTGGTWLSMDLGRGGHTSLTFEKPIKSKSGKLCLSITTRTGPVDANSRY
CRISP1_b1_CUPSA         VEGINRWIGKISTGGTWLSMDLGRGGHTSLTFEKPIKSKSGKLCLSITTRTGPVDASSRY
sp_A9QQ26_LYCSI         VEGVDRWIRNVTTGGTWLNTYLGNYGHTVIEVRSAHHLQVRQA--------MPGHQDP--
CRISP1_VIRFA            VDGYDAWSITKDVGGSRLVMNMGMGWTSTLHFTSPIKSKSGDVCIKVITRKGTLKQNKTY
tr_A0A087T5V6_STEMI     ------------------------------------------------------------
tr_A0A087T5V4_STEMI     ------------------------------------------------------------

CRISP1_a1_CUPSA         TYELGGSMEETGRFLTTMVFPKADNKVKHRFHTDYFQPSEFPKNRDVKLTLSFSVPIDGQ
CRISP1_b1_CUPSA         TYELGGSMEETGRFLTTMVFPKADNKVKHRFHTDYFQPSEFPKNRDVKLTLSFSVPIDGQ
sp_A9QQ26_LYCSI         QWTHGGRTT--------LSVPAV-------------------------------RGFGGG
CRISP1_VIRFA            NYQMGGKVELENKYMRAIGFPPVDQKTEHMYQADYFQPQNYPKNKDVKLSLTFHIPG-SN
tr_A0A087T5V6_STEMI     ------------------------------------------------------------
tr_A0A087T5V4_STEMI     ------------------------------------------------------------

CRISP1_a1_CUPSA         RQYIEIKRIVAMEKDCSKQTILEGSGYSVD
CRISP1_b1_CUPSA         RQYIEIKRIVAMEKDCSKQTILEGSGYSVD
sp_A9QQ26_LYCSI         RQILNLTHLPPCRR----------------
```

```
CRISP1_VIRFA           AQYIAIHEIVAEE-----------------
tr_A0A087T5V6_STEMI    ------------------------------
tr_A0A087T5V4_STEMI    ------------------------------
```

Percent Identity Matrix - created by Clustal2.1

		1	2	3	4	5	6
1:	CRISP1_a1_CUPSA	100.00	99.33	54.67	51.81	37.45	37.75
2:	CRISP1_b1_CUPSA	99.33	100.00	54.67	51.57	37.45	37.75
3:	sp_A9QQ26_LYCSI	54.67	54.67	100.00	49.18	39.58	38.62
4:	CRISP1_VIRFA	51.81	51.57	49.18	100.00	38.27	40.16
5:	tr_A0A087T5V6_STEMI	37.45	37.45	39.58	38.27	100.00	58.87
6:	tr_A0A087T5V4_STEMI	37.75	37.75	38.62	40.16	58.87	100.00

Proteomic coverage trypsin

>CRISP1_a1_CUPSA cysteine-rich secretory protein 1 isoform a1

```
MSAPARIRNI LLVLSSVLCT LTVIQSAYCP DIYKRYSTQH SYCLGQCCNC TYYKRGVDPG LKGQILSLHN DLRNKVAAGH
SYGVDHLPRA SNMLEMVWDD ELATIAQTWL DGCRKNGDCE ECRMVSRFAV GQEIIGDFIG SEMDSQDLDN EFFSRFNALS
VFTDAHVREY SDSNGNHKTS QLLWAKTWRI GCGFLRFLDP NNGRTVVNIL LCNYGPGGCV EGEEVYKAGT VCSACPANTC
CGDGCKKHKI TNSFKGLCKV IDENLPPQGN VPHVKSGKEL FYCGFNGESD CSRAVEGINR WIGKISTGGT WLSMDLGRGG
HTSLTFEKPI KSKSGKLCLS ITTRTGPVDA NSRYTYELGG SMEETGRFLT TMVFPKADNK VKHRFHTDYF QPSEFPKNRD
VKLTLSFSVP IDGQRQYIEI KRIVAMEKDC SKQTILEGSG YSVD
```

>CRISP1_b1_CUPSA cysteine-rich secretory protein 1 isoform a2

```
MSAPARIRNI LLVLSSVLCT LTVIQSAYCP DIYKRYSTQH SYCLGQCCNC TFYKRGVDPG LKGQILSLHN DLRNKVAAGH
SYGVDHLPRA SNMLEMVWDD ELATIAQTWL DGCRKNGDCE ECRMVSRFAV GQEIIGDFIG SEMDSQDLDN EFFSRFNALS
VFTDAHVREY SDSNGNHKTS QLLWAKTWRI GCGFLRFLDP NNGRAVVNIL LCNYGPGGCV EGEEVYKAGT VCSACPANTC
CGDGCKKHKI TNSFKGLCKV IDENLPPQGN VPHVKSGKEL FYCGFNGESD CSRAVEGINR WIGKISTGGT WLSMDLGRGG
HTSLTFEKPI KSKSGKLCLS ITTRTGPVDA SSRYTYELGG SMEETGRFLT TMVFPKADNK VKHRFHTDYF QPSEFPKNRD
VKLTLSFSVP IDGQRQYIEI KRIVAMEKDC SKQTILEGSG YSVD
```

Proteomic coverage chymotrypsin

>CRISP1_a1_CUPSA cysteine-rich secretory protein 1 isoform a1

```
MSAPARIRNI LLVLSSVLCT LTVIQSAYCP DIYKRYSTQH SYCLGQCCNC TYYKRGVDPG LKGQILSLHN DLRNKVAAGH
SYGVDHLPRA SNMLEMVWDD ELATIAQTWL DGCRKNGDCE ECRMVSRFAV GQEIIGDFIG SEMDSQDLDN EFFSRFNALS
VFTDAHVREY SDSNGNHKTS QLLWAKTWRI GCGFLRFLDP NNGRTVVNIL LCNYGPGGCV EGEEVYKAGT VCSACPANTC
CGDGCKKHKI TNSFKGLCKV IDENLPPQGN VPHVKSGKEL FYCGFNGESD CSRAVEGINR WIGKISTGGT WLSMDLGRGG
HTSLTFEKPI KSKSGKLCLS ITTRTGPVDA NSRYTYELGG SMEETGRFLT TMVFPKADNK VKHRFHTDYF QPSEFPKNRD
VKLTLSFSVP IDGQRQYIEI KRIVAMEKDC SKQTILEGSG YSVD
```

>CRISP1_b1_CUPSA cysteine-rich secretory protein 1 isoform a2

```
MSAPARIRNI LLVLSSVLCT LTVIQSAYCP DIYKRYSTQH SYCLGQCCNC TFYKRGVDPG LKGQILSLHN DLRNKVAAGH
SYGVDHLPRA SNMLEMVWDD ELATIAQTWL DGCRKNGDCE ECRMVSRFAV GQEIIGDFIG SEMDSQDLDN EFFSRFNALS
VFTDAHVREY SDSNGNHKTS QLLWAKTWRI GCGFLRFLDP NNGRAVVNIL LCNYGPGGCV EGEEVYKAGT VCSACPANTC
CGDGCKKHKI TNSFKGLCKV IDENLPPQGN VPHVKSGKEL FYCGFNGESD CSRAVEGINR WIGKISTGGT WLSMDLGRGG
HTSLTFEKPI KSKSGKLCLS ITTRTGPVDA SSRYTYELGG SMEETGRFLT TMVFPKADNK VKHRFHTDYF QPSEFPKNRD
VKLTLSFSVP IDGQRQYIEI KRIVAMEKDC SKQTILEGSG YSVD
```

Supplementary Fig S1.8 Multiple sequence alignment of CRISP1_a1 and CRISP1_b1 from the venom glands of *C. salei* (CRISP1_a1 [MH766629]; CRISP1_b1 [MH766630]), *L. singoriensis* (sp_A9QQ26_LYCSI), *V. fasciatus* (CRISP1_VIRFA), and *S. mimosarum* (tr_A0A087T5V6_STEMI, tr_A0A087T5V4_STEMI). Signal peptides are marked in black characters, cysteines in white with a black box, positively charged amino acids in pink, negatively charged amino acids in blue, hydrophobic amino acids in red and polar amino acids in green.
